# Potential Complementary Modulation of Rumen Fermentation and Lipid Metabolism in Sheep: A Hypothesis Framework for Fermented Chinese Chive Juice and Black Soldier Fly-Derived Fatty Acids

**DOI:** 10.3390/vetsci13020173

**Published:** 2026-02-09

**Authors:** Kaimin Niu, Lei Wang, Yujie Lu, Zhihong Zhang, Ping Sheng, Zongpei Zhao

**Affiliations:** 1 Institute of Biomanufacturing, Jiangxi Academy of Sciences, Nanchang 330096, China; zhangzhihong@jxas.ac.cn (Z.Z.); shengping@jxas.ac.cn (P.S.); 2 School of Grain Science and Technology, Jiangsu University of Science and Technology, Zhenjiang 212100, China; wanglei-best@just.edu.cn (L.W.); luyjlyj71@just.edu.cn (Y.L.); 3 Jiangsu Provincial Engineering Research Center of Grain Bioprocessing, Zhenjiang 212100, China

**Keywords:** Chinese chive, fermentation, black soldier fly, lauric acid, sheep, rumen fermentation, lipid metabolism, complementary effects

## Abstract

Chinese chive (*Allium tuberosum*), an edible and medicinal herb, has attracted attention for its distinctive flavor and bioactive potential. Evidence indicates that fermentation can enhance its antimicrobial and antioxidant activities, and fermented preparations have been explored as feed additives to reduce reliance on antibiotics in poultry. By contrast, evidence in ruminants—particularly sheep—remains limited, and the metabolic consequences of Chinese chive-derived compounds are not yet well defined. In parallel, fatty acids from black soldier fly (*Hermetia illucens*) larvae, with lauric acid as a prominent component, have been reported to influence rumen fermentation and aspects of lipid metabolism, suggesting a possible route to improve product traits. This review synthesizes the literature on Chinese chive and BSFL fatty acids, critically appraises how their constituents may relate to sheep nutritional metabolism, and advances a hypothesis that fermented Chinese chive juice (FCCJ) could complement black soldier fly larvae-derived fatty acids (BSFL-FA) in regulating rumen fermentation, lipid metabolism, and product quality in sheep. While the literature supports plausible complementarity, direct evidence for synergy in ruminants is still scarce and should be tested through controlled in vitro screening and well-designed in vivo sheep studies.

## Simple Summary

Chinese chive (CC) shows great potential as a functional feed additive owing to its antimicrobial and antioxidant bioactivities. Concurrently, black soldier fly (*Hermetia illucens*) larvae (BSFL) are rich in lauric acid, which has great potential to modulate rumen fermentation and lipid metabolism in sheep. Hence, this review puts forward a hypothesis for using a combination of fermented CC- and BSFL-derived fatty acids to exert complementary effects on rumen fermentation, lipid metabolism, and product quality in sheep.

## 1. Introduction

The global livestock industry, particularly ruminant production systems such as sheep farming, faces multiple and interacting challenges. Global demand for animal protein continues to increase, while requirements for environmental sustainability are becoming more stringent, placing concurrent pressures on conventional production models [1]. Growth-promoting antibiotics has caused serious concerns for antimicrobial resistance [2]. Hence, exploring safe and effective antibiotic replacers has become a global target.

Furthermore, the environmental impact of ruminant farming is a major concern. Enteric methane (CH4) produced during rumen fermentation is a major source of agricultural greenhouse-gas emissions [3]. Methane not only contributes to global warming but also represents an energy loss of approximately 2% to 12% of feed intake, thereby reducing feed-utilization efficiency [3]. Accordingly, improving feed efficiency, enhancing animal product quality (e.g., tenderness, flavor, fatty-acid composition of lamb, and the nutritional value of sheep milk), and reducing the environmental footprint of production (e.g., methane emissions) remain pressing priorities for the industry [4].

In this context, a range of natural feed additives has received attention because of their perceived safety and multifunctional properties. Phytogenic feed additives (PFAs), including plant extracts and essential oils, are frequently discussed as alternatives to antibiotic growth promoters; however, their responses can be variable and dose-dependent, and practical constraints (e.g., palatability issues, intake depression, and microbial adaptation) may limit on-farm application [5,6]. These compounds contain diverse bioactive constituents that may influence animal physiology through multiple pathways.

Meanwhile, insects are increasingly considered as a sustainable feed resource. Black soldier fly (*Hermetia illucens*) larvae (BSFL) has become a research focus because they can efficiently convert organic waste, have a short life cycle, and provide high-quality protein alongside a distinctive lipid fraction [7]. Using BSFL and related products as feed ingredients may help address feed-resource constraints and support circular-agriculture models [8].

Although individual additives can be effective for specific targets, their benefits may be constrained or accompanied by drawbacks. For example, rumen microbes can adapt to certain essential oils, leading to attenuated antibacterial or methane-reducing effects over time [9]. In principle, combining additives with complementary modes of action may generate additive or synergistic responses, where the overall effect exceeds that expected from each component alone; however, such interactions require experimental demonstration [10]. A combination strategy may therefore help mitigate limitations of single additives while addressing complex production goals through multiple mechanisms. The challenges facing livestock production—antibiotic resistance, greenhouse-gas emissions, feed efficiency, and product quality—are tightly coupled, and single-mechanism interventions are often insufficient. Consistent with pharmacological and nutritional evidence, pairing active substances with complementary mechanisms may strengthen responses and slow adaptation. Thus, future work should emphasize not only the discovery of new additives but also the rational design of combinations based on mechanism, dose, and production context [11,12].

The challenges currently facing the livestock industry are interconnected: antibiotic resistance, greenhouse gas emissions, feed efficiency, and product quality. A single solution, such as using only one methane inhibitor, is often insufficient. Pharmacological and nutritional studies have shown that combining active substances with complementary mechanisms of action can enhance effects and delay the development of adaptation. Therefore, the future research direction lies not only in discovering new additives but also in how to scientifically and intelligently combine existing resources.

**Figure 1:**
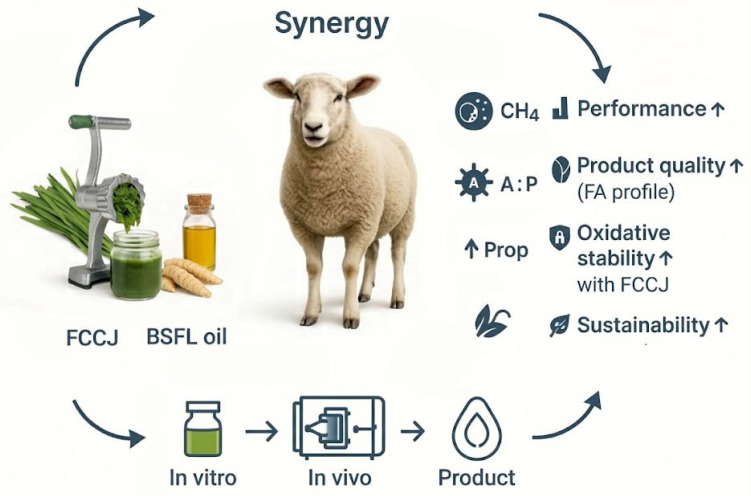
Graphical abstract of the proposed complementary effects (hypothesis) of fermented Chinese chive juice (FCCJ) and black soldier fly larvae (BSFL) oil in sheep nutrition. Using an in vitro → in vivo → product workflow, the combination is hypothesized to enhance animal performance, reduce methane (CH_4_) emissions, decrease the acetate:propionate (A:P) ratio, and increase propionate (Prop), while improving product fatty-acid (FA) profile and oxidative stability.

The present review proposes a hypothesis-driven framework that integrates FCCJ and BSFL-FA as a potential combined strategy for sheep nutrition (Figure 1). This paper aims to evaluate the existing research evidence on Chinese chive and black soldier fly fatty acids and, for the first time, construct a detailed theoretical framework explaining the potential mechanisms of their combined application in sheep nutrition, with the goal of providing a new scientific basis for simultaneously improving production performance, enhancing product quality, and achieving environmental sustainability (Figure 1).

## 2. Bioactivity and Functional Properties of Chinese Chive (*Allium tuberosum*) and Its Fermented Products

### 2.1. Key Bioactive Components: A Chemical Inventory

Chinese chive, a member of the *Allium* genus, is rich in a variety of bioactive compounds that form the basis of its unique flavor and multiple physiological functions. Modern analytical techniques have revealed its main categories of active ingredients (Table 1) [13,14].

- Organosulfur Compounds (OSCs): These are hallmark constituents of Allium plants and underpin their characteristic pungency. In Chinese chive (*Allium tuberosum*), the predominant S-alk(en)yl-L-cysteine sulfoxides are methiin (S-methyl-L-cysteine sulfoxide) and, to a lesser extent, isoalliin (S-1-propenyl-L-cysteine sulfoxide), whereas alliin (S-allyl-L-cysteine sulfoxide) occurs in lower amounts. Upon tissue disruption, alliinase converts these precursors into a mixture of thiosulfinates with strong antimicrobial activity; in *A. tuberosum* the major products are methyl- and 1-propenyl-derived thiosulfinates, and diallyl thiosulfinate (allicin) is not the dominant species [15,16].
- Polyphenolic Compounds: These mainly include flavonoids and phenolic acids. Quercetin and kaempferol, along with their glycosidic forms, are important flavonoids in Chinese chive. These compounds are the primary source of its powerful antioxidant activity [14,17,18].
- Saponins: Chinese chive contains a certain amount of steroidal and triterpenoid saponins. Saponins are known for their surface activity and various biological functions, particularly their antiprotozoal effects in ruminant nutrition [19,20].
- Chinese chive also contains general nutrients (e.g., vitamins, minerals, and dietary fiber); however, these are not the primary focus of the rumen-modulatory mechanisms discussed here [13,14].

**Table 1:** Key Bioactive Components in Chinese Chive (*Allium tuberosum*) and Their Established Biological Activities.

Compound Class	Specific Examples	Key Biological Activity	References
Organosulfur Compounds	Methiin, Isoalliin; methyl/1-propenyl thiosulfinates	Broad-spectrum antimicrobial, antiviral, anti-inflammatory	[21]
Polyphenols (Flavonoids)	Quercetin, Kaempferol	Strong antioxidant, free radical scavenging, anti-inflammatory, promotes muscle cell proliferation	[22]
Saponins	Steroidal/Triterpenoid Saponins	Antiprotozoal, cholesterol-lowering, immunomodulatory	[23]

### 2.2. Verified Biological Activities: Antimicrobial and Antioxidant Mechanisms

The biological activities of Chinese chive have been confirmed by numerous studies, with antimicrobial and antioxidant activities being the most prominent.

- Antimicrobial Activity: Studies have shown that Chinese chive extracts inhibit a variety of pathogenic microorganisms, including Gram-positive bacteria (e.g., *Staphylococcus aureus*, *Bacillus subtilis*) and Gram-negative bacteria (e.g., *Escherichia coli*, *Salmonella*), as well as some fungi [16,24,25]. One of its mechanisms of action is to disrupt the integrity of the microbial cell membrane, causing leakage of cellular contents, thereby inhibiting or killing the microorganisms [26].
- Antioxidant Activity: The high content of total phenols and flavonoids in Chinese chive is the material basis for its strong antioxidant capacity. Assays such as DPPH radical scavenging have demonstrated that Chinese chive has significant antioxidant activity, effectively scavenging excess free radicals in the body and mitigating oxidative stress damage [14,27]. Interestingly, one study found that the antioxidant activity of Chinese chive is even higher than that of the well-known garlic (*A. sativum*) [14,28].
- Other Activities: In addition to the functions mentioned above, Chinese chive also exhibits anti-inflammatory and immunomodulatory activities [14].

### 2.3. Enhanced Bioactivity Through Microbial Fermentation: Implications for Potential Complementarity

Although fresh Chinese chive exhibits multiple biological activities, evidence suggests that microbial fermentation can further enhance its efficacy. Beyond a traditional preservation approach, fermentation serves as a bioprocessing strategy that can alter the phytochemical profile of Chinese chive juice and yield preparations with strengthened biological activity [29,30].

Studies show that fermenting Chinese chive juice with specific probiotics (such as *Lactobacillus plantarum*) can significantly enhance its antimicrobial and antiviral activity against poultry pathogens [29,30]. The fermentation process alters the chemical profile of the juice, which is believed to be the reason for its enhanced bioactivity [30].

Evidence from poultry and other monogastric species provides indirect mechanistic insights; however, it is not directly transferable to ruminants due to fundamental differences in digestive physiology and rumen microbial fermentation. When FCCJ was added to broiler diets, growth performance was comparable to that of birds receiving antibiotic growth promoters. More importantly, broilers in the FCCJ group showed better health indicators, such as significantly lower levels of total cholesterol (TCHO) and aspartate aminotransferase (AST) in the blood, while the growth of potential pathogens in the gut was significantly inhibited [29,30,31]. Studies in laying hens also found that adding FCCJ could improve the storage performance of eggs and delay lipid oxidation, which is directly attributed to its enhanced antioxidant capacity [32]. Therefore, these findings should be interpreted as indirect support rather than proof of efficacy in ruminants.

## 3. Black Soldier Fly (*Hermetia illucens*) Larvae: A Novel Source of Metabolically Active Fatty Acids

### 3.1. Unique Lipid Composition: Focus on Lauric Acid

*H. illucens* larvae are a sustainable feed resource whose nutritional value lies not only in their high protein content but also in their unique and physiologically important fats. The crude fat content of *H. illucens* larvae can be as high as 40–50% of the dry matter, with its fatty acid composition being dominated by saturated fatty acids (SFAs) [33,34].

A notable characteristic of BSFL lipids is their high content of lauric acid (C12:0), a medium-chain fatty acid (MCFA). Depending on rearing substrate, lauric acid can account for 40–60% of total fatty acids in *H. illucens* larvae and, in some reports, may exceed 76% [35,36]. This variation reflects differences in substrate, larval stage/age, and downstream extraction or processing; therefore, these conditions should be reported to support meaningful comparisons across studies. The lauric-acid level is comparable to, and in some cases higher than, that of commonly used lauric-acid-rich oils such as coconut and palm kernel oil (Table 2). Accordingly, *H. illucens* larvae fat should be viewed not only as an energy source but also as a potentially practical carrier of lauric acid for applications aimed at modulating rumen microecology.

### 3.2. Biological Roles of Medium-Chain Fatty Acids in Animal Nutrition

Medium-chain fatty acids (MCFAs) such as lauric acid play a distinct role in animal nutrition because of their chemical structure and metabolic handling.

Potent Antimicrobial Activity: Lauric acid and its derivatives (such as monolaurin) exhibit broad-spectrum antimicrobial activity, with particularly strong inhibitory effects against Gram-positive bacteria [37]. Mechanistically, these compounds may act in part as surfactant-like molecules that disrupt microbial cell membranes and compromise membrane integrity.

Unique Metabolic Pathway: Unlike long-chain fatty acids, MCFAs are more easily absorbed in the digestive tract and are transported directly to the liver via the portal vein, where they are rapidly oxidized for energy rather than being preferentially stored as body fat [38].

Rumen Fermentation Regulation: In ruminants, MCFAs are recognized as rumen fermentation modulators. They may inhibit methanogenic archaea and protozoa and thereby modulate rumen fermentation, although responses can be dose- and diet-dependent [39].

In summary, the true value of black soldier fly larvae fat lies in its function as an ingredient that provides a high concentration of lauric acid with specific biological activity to the rumen, thereby offering a new tool for the precise regulation of the rumen microbial ecosystem.

## 4. Regulation of Ruminant Metabolism: Mechanisms and Evidence

Although direct studies on feeding Chinese chive to sheep are scarce, a scientific inference of its potential mechanisms of action can be made by analyzing studies on the effects of its key active components from other *Allium* plants and related plant secondary metabolites on ruminants.

### 4.1. Effects of Bioactive Substances from Allium Plants on Rumen Fermentation and Methanogenesis

When the active components of Chinese chive enter the complex microbial ecosystem of the rumen, they directly interact with the microbial community, thereby regulating rumen fermentation.

- Role of Saponins: The saponins in Chinese chive are effective antiprotozoal agents. Their mechanism of action involves forming irreversible complexes with sterols in the protozoal cell membrane, which disrupts membrane integrity and leads to cell lysis and death, a process known as “defaunation” [39,40]. Since protozoa are major predators of bacteria in the rumen, their removal can reduce the engulfment of beneficial bacteria, potentially increasing the efficiency of microbial protein synthesis and its flow to the lower digestive tract. More importantly, protozoa have a symbiotic relationship with methanogenic archaea and are one of the main providers of the hydrogen (H_2_) required by methanogens. Therefore, inhibiting protozoa can indirectly reduce substrate supply, thereby lowering methane production [41].
- Role of Organosulfur Compounds: Organosulfur compounds (OSCs) from *Allium* plants like garlic and onion have been shown to have direct inhibitory activity against methanogenic archaea [15]. In vitro studies have shown that additives containing garlic extract can reduce methane production by 22% to 54%. (These values are primarily derived from in vitro systems, and in vivo responses in sheep may differ.) These compounds may act by interfering with key enzyme systems in the methanogenesis process.
- Effects on Fermentation Patterns: By selectively inhibiting certain microbial populations, *Allium* plant extracts can alter the production pattern of volatile fatty acids (VFAs). Several studies have reported that adding *Allium* plant extracts or their active components can lower the acetate-to-propionate ratio and increase the molar concentration of propionate. Propionate is the main precursor for gluconeogenesis, and an increase in its proportion means higher efficiency in converting feed energy into usable energy for the host. However, it is worth noting that high doses of additives can sometimes inhibit total VFA production or fiber digestibility, indicating that dose optimization is essential and that responses can be context-dependent [42,43].

### 4.2. Role of Black Soldier Fly Fatty Acids in the Rumen Ecosystem

The effects of black soldier fly fatty acids, particularly lauric acid, on the rumen ecosystem have been confirmed by multiple studies.

- Mechanism of Action: As a medium-chain fatty acid, lauric acid also exerts strong antimicrobial effects in the rumen. It inhibits protozoa and some bacteria (especially Gram-positive bacteria) by disrupting the physical barrier of the microbial cell membrane [37].
- Methane Reduction: Lauric acid has been investigated as a rumen modifier with potential to reduce methane in some settings; however, reported responses vary by experimental model, basal diet, and dose, and consistent methane reduction is not universally observed in vivo. A meta-analysis of multiple studies confirmed that the addition of lauric acid significantly reduces in vitro methane production, mainly due to its inhibitory effect on methanogen and protozoa populations [37]. Because robust respiration-chamber measurements in sheep remain limited across published BSFL-FA studies, specific in vivo reduction magnitudes should be stated cautiously unless directly supported by sheep data [44].
- Effects on Fermentation Patterns: The effect of lauric acid on VFAs is clearly dose-dependent. At low to moderate inclusion levels, black soldier fly fat may increase total VFA concentration and the proportion of propionate in some in vitro systems; however, higher inclusion levels can inhibit fiber-degrading microbes and reduce total VFA production and dry matter digestibility, indicating a narrow and context-dependent dose window [45]. This phenomenon reveals that in practical applications, the dosage must be precisely controlled to find a balance between methane reduction and maintaining efficient fermentation (Table 3).

### 4.3. Downstream Effects on Host Metabolism and Product Quality

Changes in rumen fermentation directly affect the composition of nutrients entering the small intestine, which in turn has a profound impact on the host’s overall metabolism and the quality of the final products (meat and milk).

- Meat/Milk Quality: The biohydrogenation of dietary unsaturated fatty acids by rumen microbes determines the types of fatty acids ultimately absorbed by the small intestine. By modulating rumen microbes with additives, the biohydrogenation pathways can be altered, thereby affecting the fatty acid composition in tissues (muscle, fat) and milk [49]. For example, adding black soldier fly larvae to the diet of monogastric animals (poultry) resulted in a several-fold increase in the lauric and myristic acid content of their meat [50]. However, because medium-chain fatty acids (e.g., C12:0) in ruminants are transported via the portal vein and are preferentially oxidized in the liver rather than deposited in tissues, the extent of C12:0 enrichment in lamb or milk fat is limited and unlikely to reach the several-fold increases reported for monogastric species. Instead, BSFL-FA may modulate rumen fermentation and biohydrogenation patterns; however, meaningful enrichment of MUFAs/PUFAs in ruminant products generally requires co-supplementation with unsaturated fat sources or protection strategies.
- Muscle Growth: As some research suggests that flavonoids may play a role in muscle cell development, the flavonoid content of chives could potentially contribute to improved meat production, though this requires direct verification in livestock [51].

## 5. Hypothesized Complementary Interactions: Combining Fermented Chinese Chive Juice and Black Soldier Fly-Derived Fatty Acids

**Figure 2:**
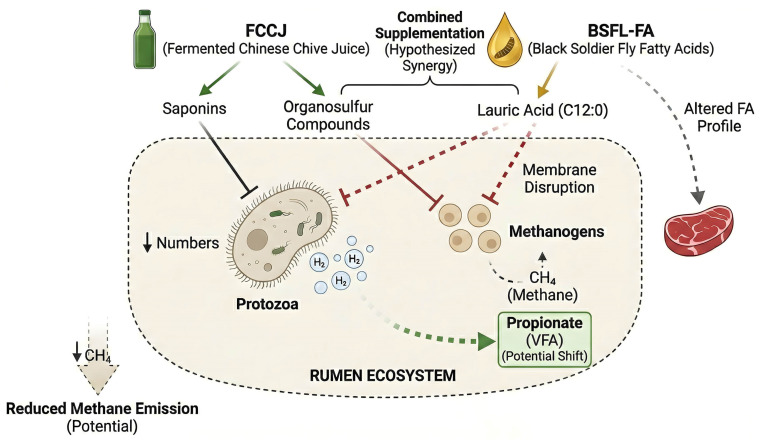
Hypothesized complementary mechanisms of fermented Chinese chive juice (FCCJ) and black soldier fly larvae-derived fatty acids (BSFL-FA) in the rumen ecosystem. FCCJ bioactives (e.g., saponins and organosulfur compounds) and BSFL-FA (with lauric acid as a representative medium-chain fatty acid) are proposed to modulate rumen microbial ecology, including potential reductions in protozoa and effects on membrane-sensitive microbial niches. These changes may redirect hydrogen use away from methanogenesis toward propionate as an alternative hydrogen sink, thereby lowering enteric methane (CH_4_) emission potential. BSFL-FA may also influence product fatty-acid (FA) profiles via altered lipid supply and deposition. Solid arrows denote mechanisms supported for individual components in rumen-relevant studies, whereas green dashed arrows represent the hypothesized redirection of metabolic hydrogen toward propionate synthesis, a pathway requiring specific validation in sheep.

Based on the preceding analysis of the individual properties of the two additives, this section innovatively proposes a core hypothesis: combining the complex bioactivities of FCCJ with the targeted metabolic regulatory functions of BSFL-FA may produce meaningful additive or synergistic effects, but this remains a testable hypothesis that requires controlled validation in sheep (Figure 2). This combination is not a simple functional overlap but is expected to achieve a comprehensive improvement that surpasses the effects of the individual components through complementary mechanisms and potentially additive (or synergistic) interactions (Table 4).

### 5.1. Potential Mechanisms for Complementary Regulation of Rumen Microecology

The combination of FCCJ and BSFL-FA is expected to form a multi-target, multi-pathway regulatory network at the rumen level.

- Complementary Antimicrobial Spectrum: The active substances from FCCJ and BSFL-FA differ in chemical structure and mode of action (e.g., enzyme inhibition versus cell-membrane disruption), and may therefore target partially distinct microbial populations. Such non-overlapping targets could broaden the overall antimicrobial spectrum relative to a single component and more effectively suppress microbes that are undesirable or metabolically inefficient in the rumen.
- Multi-pronged Methane Reduction Strategy: This combination presents a multi-faceted attack on the methanogenesis process, which may be more effective and less prone to microbial adaptation than single-mechanism inhibitors.
  1. Dual Defaunation Effect: The saponins in FCCJ and the lauric acid in BSFL-FA both effectively inhibit protozoa, cutting off a major hydrogen source for methanogens from two angles.
  2. Direct Inhibition of Methanogens: The organosulfur compounds in FCCJ and the lauric acid in BSFL-FA can directly act on methanogenic archaea through different mechanisms to inhibit their activity.
  3. Competition for Hydrogen Utilization: Both additives are likely to promote the production of propionate, and the synthesis of propionate is itself a hydrogen-consuming pathway. This creates competition for metabolic hydrogen with the methanogenesis process, thereby directing hydrogen towards a pathway more beneficial for the host’s energy utilization.

### 5.2. Potential Complementary Effects on Health and Product Quality

The potential value of combining FCCJ and BSFL-FA is not only reflected within the rumen but may also extend to host health and product quality; however, these combined outcomes remain to be validated in sheep.

- Potential Antioxidant-Mediated Protection of Lipids: This is a plausible complementary mechanism that warrants validation. Given its lauric-acid-rich and largely saturated profile, BSFL-FA is more likely to increase the contribution of medium-chain saturated fatty acids (e.g., C12:0, C14:0) to the absorbed lipid pool. If the goal is to enrich MUFAs/PUFAs in tissues or milk, co-supplementation with unsaturated fat sources (e.g., flaxseed or fish oil) or protection strategies is generally required. In that context, the potent antioxidants in FCCJ may help protect these more oxidation-prone PUFAs by improving oxidative stability of meat and milk fat [52]. At the same time, the potent antioxidants like flavonoids in FCCJ, after being absorbed by the animal, can exert systemic antioxidant effects in the body. Thus, a “protection and enhancement” model is formed: BSFL-FA is responsible for optimizing the fatty acid profile, while the antioxidants from FCCJ protect these newly formed, more easily oxidized valuable fatty acids. This potential interaction may go beyond a simple addition in the rumen and directly affects the economic value and consumer acceptance of the final product.
- Potential Promotion of Muscle Growth and Meat Quality: FCCJ’s flavonoids may promote muscle cell growth, while BSFL-FA can influence intramuscular fat deposition, which is crucial for meat tenderness and flavor. Combining these effects may contribute to improvements in muscle development and meat quality; however, this remains speculative without controlled in vivo validation.
- Enhanced Health and Immune Function: The anti-inflammatory and antioxidant properties of FCCJ, combined with the antimicrobial activity of both additives, can provide a comprehensive health support system for the animals, potentially enhancing the resistance of sheep to environmental stress (such as weaning, transport) and diseases.

## 6. Conclusions and Future Research Perspectives

### 6.1. Evidence Synthesis and Hypothesis Confirmation

This review has examined the research progress on Chinese chive (especially FCCJ) and black soldier fly fatty acids. Existing evidence indicates that fermentation of Chinese chive significantly enhances its biological activity and has demonstrated great potential as an antibiotic alternative in poultry. Simultaneously, lauric acid-rich fat from black soldier fly larvae shows potential to modulate rumen fermentation and to influence methane emissions and product fatty-acid profiles in ruminants, though robust in vivo confirmation—especially in sheep—remains needed.

The core innovation of this review is the first integrative proposal of a scientific concept to combine the diverse bioactivities of fermented Chinese chive juice with the targeted metabolic regulatory functions of black soldier fly fatty acids for application in sheep farming. Theoretical analysis suggests that the two have plausible synergistic potential in regulating the rumen microecology, enhancing antioxidant and immune functions, and potentially improving lipid metabolism and muscle growth. This combination may offer new, green solutions to the challenges faced by modern livestock farming, especially the sheep industry.

### 6.2. Key Knowledge Gaps and Future Research Directions

Despite the promising outlook, moving this proposed combined strategy from theory to practice still faces numerous challenges, and future research is urgently needed to fill the following key knowledge gaps:

- Lack of Direct In Vivo Validation: Currently, systematic in vivo studies on directly feeding Chinese chive (fermented or not) to ruminants like sheep are extremely scarce. Its efficacy in ruminants is mainly inferred from knowledge of its active components and from poultry research. This is the weakest link in the current theoretical framework.
- Dose–Effect Relationship and Optimal Ratio: Both FCCJ and BSFL-FA are complex mixtures, and the interactions between their components (additive, synergistic, antagonistic, or unrelated) are difficult to predict. Determining the optimal dosage and ratio for both individual and combined use is key to achieving the desired effects and avoiding potential negative impacts at high doses (such as inhibiting digestibility).
- Long-term Effects and Adaptation: Long-term feeding trials that span meaningful portions of the production cycle are needed to assess the persistence of effects and to determine whether rumen microbes adapt to the composite additive.
- Diet Interaction Effects: The magnitude and direction of responses may depend on basal-diet composition (e.g., high-concentrate versus high-forage diets), requiring studies tailored to representative feeding systems.
- Economic Feasibility Assessment: Cost–benefit analyses should weigh acquisition and processing costs (e.g., Chinese chive fermentation, black soldier fly rearing, and oil extraction) against practical benefits (e.g., weight gain, feed conversion, and potential value addition).

### 6.3. Roadmap for Product Development and In Vivo Validation

To advance the development of this hypothesized combined strategy, future research should follow a stepwise path from in vitro screening to in vivo validation and product development:

Step One: In Vitro Screening and Preliminary Mechanism Exploration: Rumen simulation approaches (e.g., RUSITEC) can be used to screen FCCJ and BSFL fat (or purified oil) across ratios and dosages. By quantifying methane output, VFA profiles, ammonia-N, and key microbial groups (e.g., protozoa, methanogens, and fiber-degrading bacteria), researchers can preliminarily evaluate interaction patterns and identify candidate formulations for subsequent testing.

Step Two: Systematic In Vivo Feeding Trials: Sheep should be used as the model to conduct rigorously controlled in vivo trials. These studies should comprehensively evaluate performance outcomes, rumen fermentation parameters, microbial shifts, and product-relevant traits, thereby determining whether the combined strategy delivers additive or synergistic benefits under practical dietary conditions.

- Production Performance: Feed intake, average daily gain, feed conversion ratio.
- Rumen Fermentation and Digestion: Rumen fluid pH, VFAs, ammonia-N, microbial community composition, apparent nutrient digestibility.
- Environmental Impact: Accurately measure methane emissions using respiration chambers.
- Health Status: Blood biochemical parameters, immune and antioxidant indicators.
- Product Quality: Detailed analysis of the fatty acid and amino acid profiles of lamb and sheep’s milk, assessment of meat quality parameters such as tenderness and color, and oxidative stability (shelf-life) tests.

Step Three: Technology Optimization and Industrial Application: Optimize the fermentation process for Chinese chive and the farming and oil extraction techniques for black soldier flies to reduce costs and improve the yield and stability of active components. Develop standardized composite additive products with stable properties that are easy to store and feed (e.g., powders, granules, microcapsules). Strengthen collaboration with feed companies and farms to promote the translation and application of research findings.

In conclusion, the combined application of fermented Chinese chive juice and black soldier fly fatty acids provides a plausible rationale for improving the nutrition, health, and product quality of sheep, and even for promoting the sustainable development of the livestock industry. Although current research is still in the early exploratory stage, with the deepening of basic research and breakthroughs in applied technology, this innovative strategy may contribute to guiding future research to solving the bottleneck problems of the industry. Overall, the framework developed here is hypothesis-driven and requires controlled in vivo validation in sheep before practical recommendations can be made.

## Figures and Tables

**Table 2:** Comparative Fatty Acid Composition of Black Soldier Fly Larvae Fat, Coconut Oil, and Palm Kernel Oil (as a percentage of total fatty acids) [33,34,35,36,37].

Fatty Acid	Common Name	BSFL Fat (%)	Coconut Oil (%)	Palm Kernel Oil (%)
C12:0	Lauric acid	40–60	45–53	46–48
C14:0	Myristic acid	6–10	16–21	15–17
C16:0	Palmitic acid	12–22	7–10	8–9
C18:1	Oleic acid	10–25	5–10	14–15
C18:2	Linoleic acid	2.5–12.5	1–3	2–3

**Table 3:** Summary of reported effects of Allium plant active components and lauric acid/BSFL-derived fat on ruminant fermentation parameters (with experimental model and dose).

Additive Category	Parameter	Experimental Model	Typical Dose/Concentration	Effect	Notes/Limitations	Key Refs
*Allium* Bioactives	Methane (CH_4_)	In vitro (rumen batch culture: garlic juice/powder—fermentation & gas production); in vivo (Simmental calves: Allium mongolicum Regel)	In vitro: garlic juice 0.5–1.0 mL/100 mL (≈5–10 mL/L) and/or garlic powder (as tested); In vivo: 200–800 mg/kg BW/day	Reduced	In vivo CH_4_ reduction demonstrated in calves; some in vitro Allium studies infer methane-mitigation potential from fermentation shifts, although direct CH_4_ measurement is available in some gas-production systems.	[42,46]
	Protozoa	Mechanistic + applied rumen evidence from saponin-containing plant materials (review-based synthesis)	Varies by saponin source/preparation (not standardized across studies)	Reduced	Defaunation mechanism is well-established for saponins; direct protozoa-count data for Chinese chive-derived preparations remain limited.	[19,20]
	Total VFA	In vitro (batch culture: garlic); in vivo (Simmental calves: Allium mongolicum Regel)	As above (in vitro garlic preparations; in vivo 200–800 mg/kg BW/day)	Variable/dose-dependent	Often maintained or increased at moderate inclusion; response depends on dose and basal diet.	[42,46]
	Acetate:Propionate	In vitro (batch culture: garlic); in vivo (Simmental calves: Allium mongolicum Regel)	As above	Reduced	Consistent with a shift in H_2_ use toward propionate as an alternative sink.	[42,46]
	Ammonia-N	In vitro (batch culture: garlic preparations)	Garlic powder or garlic juice 0.5 mL/100 mL (per study designs)	Reduced	Ammonia reduction is reported in vitro with garlic preparations; in vivo ammonia response for Allium mongolicum is not explicit in the abstract.	[42]
Lauric Acid/BSFL Fat	Methane (CH_4_)	Meta-analysis (MCFA; in vitro & in vivo) + in vitro gas production (BSFL oil; BSF larvae meal) + in vivo (Thai-indigenous steers; methane estimation)	Oil: 1–4% DM (in vivo)/2–6% DM (in vitro); Larvae meal: 5–15% DM (in vitro)	Reduced (consistent in vitro); variable (in vivo)	Meta-analysis shows CH_4_ decreases with increasing MCFA dose; steer trial reports a linear decline in CH_4_ estimate with 1–4% oil; diet and dose influence in vivo magnitude.	[40,44,47,48]
	Protozoa	Meta-analysis (MCFA; in vitro & in vivo) and in vivo (Thai-indigenous steers)	Oil: 1–4% DM	Reduced	Protozoa respond negatively (dose-related) to MCFA; steer trial reports protozoa decreasing with increasing BSFL oil inclusion.	[40,44]
	Total VFA	In vivo (steers) + in vitro (goat-diet fermentation with larvae meal; BSFL oil with R:C ratios)	Oil: 1–4% DM (in vivo)/2–6% DM (in vitro); Larvae meal: 5–15% DM (in vitro)	Dose-dependent	Moderate inclusion can maintain fermentation; higher larvae meal (e.g., 15% DM) may reduce CH_4_ but also depress VFA in vitro.	[44,47,48]
	Acetate:Propionate	In vivo (steers) and in vitro (BSFL oil with R:C ratios)	Oil: 1–4% DM (in vivo)/2–6% DM (in vitro)	Reduced/no effect	Often associated with higher propionate proportion; steers showed higher propionic acid at 2% oil in one sampling time.	[44,48]
	Dry Matter Digestibility	Meta-analysis (MCFA; digestibility outcomes) + in vivo (steers: BSFL oil) + in vivo (steers: lauric acid, high-grain diet)	Oil: 1–4% DM (in vivo; quadratic response); Lauric acid: high-grain steer model (dose per study); higher MCFA doses reduce fibre digestibility (meta-analysis)	Dose-dependent (can decrease at higher dose)	Meta-analysis reports fibre digestibility declines with increasing MCFA dose; steer trial with BSFL oil shows a quadratic DM/OM digestibility response; lauric acid evidence is from a high-grain steer model.	[7,40,44]

**Table 4:** Hypothesized Mechanisms of Potential Additive (or Synergistic) Interactions between Fermented Chinese Chive Juice and Black Soldier Fly Fatty Acids in Sheep.

Target Area	Mechanism of Fermented Chinese Chive Juice (FCCJ)	Mechanism of Black Soldier Fly Fatty Acids (BSFL-FA)	Expected Outcome (Hypothesized)
Rumen Microecology	Provides a complex of antimicrobial substances (OSCs, flavonoids, saponins, and organic acids) to modulate the microbial community structure. †	Provides a high concentration of lauric acid, potently inhibiting Gram-positive bacteria and protozoa. †	A broader and more potent antimicrobial effect, effectively optimizing the rumen microbial ecosystem.
Methane Reduction	Saponins for defaunation †; OSCs may directly inhibit methanogens. ‡	Lauric acid for defaunation † and may directly inhibit methanogens. ‡	Potential additive (or synergistic) inhibition of methane production through multiple targets and pathways, resulting in a more lasting and significant effect.
Product Quality	Flavonoids may promote muscle growth ‡; antioxidants protect tissues. †	Modulates rumen biohydrogenation†; may alter product fatty-acid composition and may require co-supplementation with unsaturated fat sources or protection strategies. ‡	Improved meat/milk quality via microbial modulation and antioxidant protection, including enhanced oxidative stability and potential nutritional value.
Animal Health	Antioxidant †, may exert anti-inflammatory ‡ and immunomodulatory effects. ‡	Antimicrobial †, antiprotozoal †; may improve gut health. ‡	Comprehensive enhancement of the animal’s stress resistance and disease resilience.

Note: † Evidence-supported in rumen-relevant studies/established mode of action. ‡ Proposed/plausible; requires validation in sheep (endpoint-specific).

## Data Availability

No new data were created or analyzed in this study. Data sharing is not applicable to this article.
